# Relationships between Bloom’s taxonomy, judges’ estimation of item difficulty and psychometric properties of items from a progress test: a prospective observational study

**DOI:** 10.1590/1516-3180.2019.0459.R1.19112019

**Published:** 2020-04-22

**Authors:** Pedro Tadao Hamamoto, Eduardo Silva, Zilda Maria Tosta Ribeiro, Maria de Lourdes Marmorato Botta Hafner, Dario Cecilio-Fernandes, Angélica Maria Bicudo

**Affiliations:** I MD, PhD. Physician, Department of Neurology, Psychology and Psychiatry, Universidade Estadual Paulista (UNESP), Botucatu (SP), Brazil.; II BSc. Statistical Manager, Edudata Informática, São Paulo (SP), Brazil.; III MD. Assistant Professor, Faculdade de Medicina de Marília (FAMEMA), Marília (SP), Brazil.; IV MD, MSc. Assistant Professor, Faculdade de Medicina de Marília (FAMEMA), Marília (SP), Brazil.; V PhD. Researcher, Department of Medical Psychology and Psychiatry, Universidade Estadual de Campinas (UNICAMP), Campinas (SP), Brazil.; VI MD, PhD. Associate Professor, Department of Pediatrics, Universidade Estadual de Campinas (UNICAMP), Campinas (SP), Brazil.

**Keywords:** Psychometrics, Educational measurements, Medical education, Progress testing, Standard setting, Bloom’s taxonomy, Angoff method

## Abstract

**BACKGROUND:**

Progress tests are longitudinal assessments of students’ knowledge based on successive tests. Calibration of the test difficulty is challenging, especially because of the tendency of item-writers to overestimate students’ performance. The relationships between the levels of Bloom’s taxonomy, the ability of test judges to predict the difficulty of test items and the real psychometric properties of test items have been insufficiently studied.

**OBJECTIVE:**

To investigate the psychometric properties of items according to their classification in Bloom’s taxonomy and judges’ estimates, through an adaptation of the Angoff method.

**DESIGN AND SETTING:**

Prospective observational study using secondary data from students’ performance in a progress test applied to ten medical schools, mainly in the state of São Paulo, Brazil.

**METHODS:**

We compared the expected and real difficulty of items used in a progress test. The items were classified according to Bloom’s taxonomy. Psychometric properties were assessed based on their taxonomy and fields of knowledge.

**RESULTS:**

There was a 54% match between the panel of experts’ expectations and the real difficulty of items. Items that were expected to be easy had mean difficulty that was significantly lower than that of items that were expected to be medium (P < 0.05) or difficult (P < 0.01). Items with high-level taxonomy had higher discrimination indices than low-level items (P = 0.026). We did not find any significant differences between the fields in terms of difficulty and discrimination.

**CONCLUSIONS:**

Our study demonstrated that items with high-level taxonomy performed better in discrimination indices and that a panel of experts may develop coherent reasoning regarding the difficulty of items.

## INTRODUCTION

Assembling a knowledge test can be a challenging task, especially with regard to calibrating the difficulty of the test. Although many studies have addressed how useful experts’ opinions can be, their predictions of the difficulty is often different from what the students perceive. This uncertainty relates to the multiple factors involved in the cognitive process that is necessary for answering a question and to the tendency of item-writers to overestimate students’ performance.^1^^,^^2^ Questions can require lower or higher levels of cognitive processing, depending on whether students have to recall, minimally understand or apply their knowledge. Although studies have investigated experts’ predictions and the requirements for cognitively processing the items, little attention has been paid to the combination of these two factors. Knowing whether there are relationships between the type of cognitive processing that the item requires, experts’ predictions and the difficulty of the items may help experts to predict the difficulty of knowledge tests better.

Bloom’s taxonomy of educational objectives was designed to classify the learning objectives, skills and abilities that are expected from learners at the end of an educational program.^3^^,^^4^ Educational objectives may range from memorization of knowledge to creation of new knowledge in an increasingly complex and hierarchical fashion.^3^^,^^5^ Within this framework, cognitive processing is represented as a cumulative hierarchy that is made up of lower and higher levels of acquired knowledge. There are two low levels, which relate to remembering and minimally understanding the knowledge. There are two intermediate levels (third and fourth levels), which relate to applying the knowledge to a new situation and making connections between ideas (analyses). There are two high levels, which relate to justifying decisions (evaluations) and creation of new knowledge. In theory, mastery of lower levels is required in order to attain higher levels.

Questions that assess higher levels of complexity of knowledge are difficult to produce, and there is a debate regarding whether multiple-choice questions have the capacity to assess higher levels of complexity, i.e. situations of creation of new knowledge.^6^ More importantly, higher-order cognitive processing has been shown to improve students’ knowledge retention, compared with low-order cognitive processing. Additionally, medical practice requires the use of higher-order cognitive processing more than lower-order processing. Although there is a trend within medicine towards assessing students at higher levels of cognitive processing, little attention has been paid to Bloom’s taxonomy when setting pass/fail scores.

Setting pass/fail scores is the main concern in educational assessment.^7^^,^^8^ There are two main categories of procedures for setting standards: norm-referenced (relative) and criterion-referenced (absolute). Relative methods take the results from the test into account to set the standards. They help rank the examinees but may lead to a large variation in the cutoff scores and are poorly accepted in some cultures. Absolute methods are widely used worldwide, but they face several criticisms because they lead to large variation in failure rates and do not consider the different difficulties between different exams.^9^^,^^10^

One example of a criterion-referenced method that is often used within medical education is the Angoff method. In this method, the judges of the examination estimate the percentage of borderline examinees who will respond correctly to the test items. The judges’ estimates are then averaged for each item, and the cutoff is set as the sum of the averages.^11^

Progress tests have been used in Brazilian schools for more than fifteen years.^12^^,^^13^^,^^14^ They have been gaining greater attention over the last five years because of the Brazilian Association of Medical Education’s efforts to improve the quality of medical students’ evaluations throughout the country.^15^ Therefore, progress tests give rise to a good opportunity for studying the psychometric properties of assessment items.

## OBJECTIVE

Although some studies have analyzed the application of Bloom’s taxonomy to test items^16^ and the utility of Angoff methods using standard settings,^17^^,^^18^ the relationship between these two has not been extensively examined. In the current study, we investigated the relationships between the exam judges’ estimates (through an adaptation of the Angoff method) and the classification of the difficulty and discrimination levels of items, using Bloom’s taxonomy in a progress test setting.

## METHODS

### Study design

For this prospective observational study, data from the 2018 progress test from a consortium of ten Brazilian medical schools, mainly in the state of São Paulo, Brazil, were examined. Our examination of the progress test was designed to assess the knowledge that final-year medical students should have, in order to provide feedback to medical students and institutions.^15^ All the students at these ten schools underwent the same test once a year, on the same day, at the same time. The students had four hours to complete the test, and after two hours had elapsed, they could use the question booklet of the test for self-study purposes. Written feedback with commentary and bibliographic references for each item was provided a few days after the test.

A blueprint for the progress test was developed by the consortium, consisting of six fields of knowledge: basic science, internal medicine, pediatrics, surgery, obstetrics and gynecology, and public health. Every year, the coordinators of the progress test create a set of orders for items that address the blueprint. Each school is represented at the meetings by an academic staff member. This representative is responsible for the exchange of information between his school and the others, as well as for delivering the orders to his colleagues, who will be responsible for writing the required items. A single order from the coordinators might therefore consist of up to ten written items. Afterwards, several specialists from the consortium schools hold a meeting to select the items that will make up the final exam: 20 items for each field, thus totaling 120 multiple-choice items, each presenting four alternative responses. Any unused items are stored in a database.

### Bloom’s taxonomy classification of the items

The items were classified in accordance with the levels of cognitive domains that were proposed by Bloom, as revised by Anderson and Krathwohl.^5^ Here, items focusing on remembering and developing minimal understanding of knowledge were classified as the lowest taxonomy level; items focusing on knowledge application and analysis were classified as the intermediate taxonomy level; and items focusing on synthesis and evaluation were classified as the highest taxonomy level. These items were classified by two experts, who classified the items in accordance with their use in tests over the past five years.

### Angoff adaptation

In this study, the panel of experts was asked to set the expected difficulty for each item selected. The difficulty would be estimated by considering the performance of a sixth-year medical student. In the original use of the Angoff method, the expected percentage of correct answers among the examined population was ascertained.^19^ Here, we asked the experts to classify the items as follows: difficult (expectation that more than 80% of the answers would be incorrect), medium (expectation that 40% to 80% would be incorrect), and easy (expectation that less than 40% would be incorrect). The expected level of difficulty of the items was developed based on an agreement that was reached after a discussion among the judges.

### Statistical analysis

A specialized institution marked the tests and performed psychometric analysis on the items by focusing on their difficulty, the discrimination index and biserial correlation. This last aspect will not be discussed further in the present study. For the purpose of the present study, test responses that consisted of guessing constant answers were excluded from the analysis (i.e. proportion of correct answers < 25%). We only used the data from the sixth-year students at the ten medical schools.

As described above, items with a difficulty index greater than 0.8 were considered difficult, items with indices lower than 0.4 were considered easy and items with indices between 0.4 and 0.8 were considered medium.

The normality of the data was tested using the Shapiro-Wilk test. The differences in mean values were tested using single-factor analysis of variance (ANOVA) followed by the Tukey post-test for the parametric data; or using the Kruskal-Wallis test followed by the Dunn test for the nonparametric data. Correlations between the different data were made using the Spearman correlation test. We set the statistical significance level at a P-value of 0.05.^20^ The statistical analyses were performed using the Statistical Package for the Social Sciences (SPSS), version 24.0, and the BioEstat software, version 5.0.

### Ethical considerations

Since we dealt with secondary data and no student was identified, ethics committee approval was not necessary.

## RESULTS

A total of 4,596 students participated in the test (94.1% of the total population), from which 4,563 were included in the general psychometric analysis. Of these, 771 students were in their sixth year (Table 1). One item relating to obstetrics and gynecology was invalidated due to inconsistent answers, and therefore, 119 items were analyzed.

**Table 1.** Summary of the students who sat the examination, according to school and undergraduate year

School	1^st^ year	2^nd^ year	3^rd^ year	4^th^ year	5^th^ year	6^th^ year
UNICAMP	117	112	113	115	122	125
UNESP	90	91	85	90	83	88
USP-RP	93	96	93	92	95	111
USP-BA	59	0	0	0	0	0
UNIFESP	116	124	115	111	117	127
UFSCAR	39	41	40	33	37	43
FAMEMA	79	78	71	80	75	79
FAMERP	74	81	74	76	79	62
UEL	79	79	73	78	61	72
FURB	72	82	76	70	69	64
Total	818	784	740	745	738	771

UNICAMP = Universidade Estadual de Campinas; UNESP = Universidade Estadual Paulista; USP-RP = Universidade de São Paulo-Ribeirão Preto; USP-BA = Universidade de São Paulo-Bauru; UNIFESP = Universidade Federal de São Paulo; UFSCAR = Universidade Federal de São Carlos; FAMEMA = Faculdade de Medicina de Marília; FAMERP = Faculdade de Medicina de São José do Rio Preto; UEL = Universidade Estadual de Londrina; FURB = Fundação Universidade Regional de Blumenau.

### Bloom’s taxonomy

**Figure 1.**
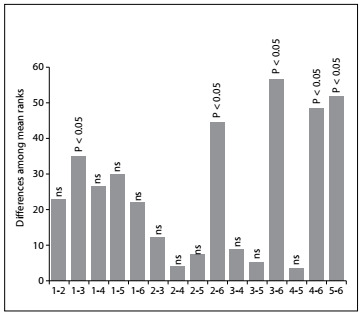
Differences among the mean ranks of the fields of knowledge, in accordance with the classification of Bloom’s taxonomy. 1: basic sciences; 2: internal medicine; 3: pediatrics; 4: surgery; 5: obstetrics and gynecology; 6: public health. ns: non-significant. Public health was significantly different from internal medicine, pediatrics, surgery and obstetrics and gynecology. Pediatrics was also significantly different from basic sciences. Overall, P < 0.0001.

The 119 items were classified using Bloom’s taxonomy. Of these, 52 (43.7%) had high-level taxonomy, 32 (26.9%) had medium-level taxonomy and 35 (29.4%) had low-level taxonomy. More than 50% of the items relating to internal medicine, pediatrics, surgery and obstetrics and gynecology were classified as presenting high-level taxonomy, whereas most of the items relating to basic sciences and public health were classified as presenting low-level taxonomy. The distribution of the items was significantly different between the fields (P < 0.001), such that public health presented higher frequency of items with low-level taxonomy, compared with internal medicine, pediatrics, surgery and obstetrics and gynecology. In addition, the distribution of items was statistically different between pediatrics and basic sciences (Figure 1). Table 2 presents the distribution of items according to their taxonomy among the fields of knowledge.

**Table 2.** Summary of psychometric properties and distribution of Bloom’s taxonomy according to the fields of knowledge of the exam

Area	Mean difficulty	Mean discrimination	High-level taxonomy	Medium-level taxonomy	Low-level taxonomy
Basic sciences	0.36	0.27	20%	35%	45%
Internal medicine	0.35	0.32	50%	35%	15%
Pediatrics	0.32	0.32	75%	15%	10%
Surgery	0.34	0.32	55%	35%	10%
Obstetrics and gynecology	0.36	0.38	63%	26%	11%
Public health	0.29	0.31	0%	15%	85%
Total	0.34	0.32	44%	27%	29%

### Item difficulty

**Figure 2.**
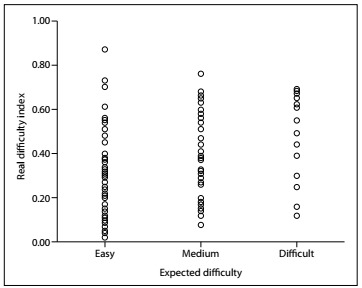
Scatter diagram illustrating the indices of real difficulty of the items, according to their classification by the panel of judges.

The panel of experts judged 62 items as easy, 41 as medium and 16 as difficult. Based on the analysis of the real difficulty of the items, 79 items were easy, 82 were medium and only one item was difficult (Figure 2). For 65 items (54%), the expected difficulty was the same as the difficulty in reality; 13 items (11%) were underestimated (i.e. they were more difficult than expected); and 41 items (34%) were overestimated (i.e. they were easier than expected). The rates of concordance between expected difficulty and difficulty in reality were 60% for basic sciences, pediatrics and public health; 50% for internal medicine and surgery; and 47% for obstetrics and gynecology.

The analysis on the difficulty of the items in reality according to the levels of difficulty set by the panel experts demonstrated mean difficulties of 0.28, 0.37 and 0.49, for items considered easy, medium and difficult, respectively. These differences were statistically significant (F = 8.604; P < 0.01): the items that were considered easy presented mean difficulty significantly lower than that of the items considered medium (P < 0.05) and the items considered difficult (P < 0.01).

Obstetrics and gynecology and basic sciences were the categories with the highest mean difficulty, followed by internal medicine, surgery, pediatrics and public health (Table 2). We did not find any significant differences between the fields of knowledge (F = 0.323; P = 0.898), although there was a trend towards public health to be considered easier.

The mean difficulties of the items classified as having low, intermediate and high-level taxonomies were 0.29, 0.34 and 0.36, respectively. We did not find any significant differences between the levels of taxonomy regarding difficulty (F = 0.993; P = 0.374), and we did not find any correlation between the taxonomy of the items and their difficulty (rho = 0.172; P = 0.06).

### Item discrimination

The mean discrimination indices were 0.38 for obstetrics and gynecology; 0.32 for pediatrics, surgery and internal medicine; 0.31 for public health; and 0.27 for basic sciences (Table 2). Although obstetrics and gynecology demonstrated a trend towards greater discrimination, we did not find any significant differences between the fields of knowledge (H = 8.734; P = 0.12).

**Figure 3.**
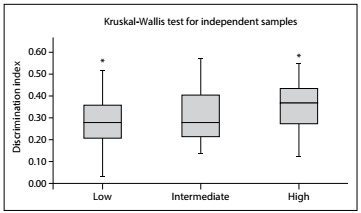
Differences between the taxonomy groups regarding mean discrimination indices. The items with low-level taxonomy had significantly lower discrimination indices than the items with high-level taxonomy (P = 0.026).

Comparison of discrimination between the items according to their taxonomy group demonstrated mean discrimination indices of 0.28, 0.31 and 0.35 for items with low, intermediate and high levels of taxonomy, respectively. A statistical difference was found between the groups with low and high levels of taxonomy (P = 0.026; Figure 3). A Spearman correlation test demonstrated that there was a positive correlation between the taxonomy of the items and their discrimination indices (rho = 0.25; P = 0.006).

## DISCUSSION

This study sought to use progress tests to investigate the relationships between the difficulties and discrimination and the judges’ estimates of exam items, through an adaptation of the Angoff method; and to classify them using Bloom’s taxonomy. Items with higher-level taxonomy had higher discrimination indices than those with lower-level taxonomy. We also found that items that were expected to be easy were indeed easier than items that were expected to be difficult.

At the end of medical school, students are expected to demonstrate high-order cognitive processes. For example, students in the initial years of training perform better in questions with lower-level taxonomy, whereas students in their final years perform better in relation to items with higher-level taxonomy.^21^ In our test, items with higher-level taxonomy predominated, which was expected because the test was designed to include vignette-based items. In addition, tests with higher-level taxonomy had better discrimination indices than tests with lower-level taxonomy. These data emphasize the need to develop tests for better discrimination of items with high-level taxonomy. In this regard, case-based questions might be more suitable for higher-order cognitive processing^12^ and consequently might be more appropriate for tests that are designed to assess the knowledge of final-year students.

Interestingly, in the field of public health, the indices of discrimination and difficulty tended to be lower. This can possibly be explained in terms of the predominance of lower-order cognitive processes that are involved in the items from this subject. These findings may relate to the characteristics of this field: students are required to have sufficient knowledge of legislation and conceptual frameworks.

Although the test was easier than estimated by the judges, the mean values for the total score were in accordance with those found in other studies on progress testing data.^22^^,^^23^^,^^24^ While the low achievement of students at the final-year level creates doubt regarding the unrealistic expectations of item writers and the quality of the items,^25^ discussion of the underestimated items can be useful for medical schools and their academic staff as a means for monitoring the educational environment.

The panel of experts demonstrated coherent reasoning in classifying the difficulty of the items. In addition, the group analysis indicated that the items that were expected to be easy presented lower mean difficulty indices than the items that were expected to be medium or difficult; while items that were expected to be medium had lower mean difficulty indices than items that were expected to be difficult (although these differences were not statistically significant).

Similarly, Kibble and Johnson found coherence between the intended and actual difficulty of the items, with a successful estimation rate of 48%.^26^ Conversely, they did not find any correlation between the taxonomy of the test items and their difficulty and discrimination indices. These authors placed doubt on the usefulness of efforts for estimating the difficulty of items and their taxonomy as a means for controlling examination difficulty. This may have been due to the tendency of the item writers to overestimate the students’ performance, and to the fact that item writers and examinees approach the same material in different ways, based on different levels of knowledge.^2^^,^^27^ Corroborating this hypothesis, Verhoeven et al. found that using recent graduates as judges for setting progress testing standards had good reliability and credibility, and subsequently found that the data from recent graduates were more credible than data from item writers, regarding their estimates as judges.^17^^,^^18^

The present study had some limitations: firstly, we used only one edition of the progress test, and the number of items analyzed was limited. Continuous monitoring of the items applied by our consortium may strengthen our findings. Secondly, this was the first time that we had used the Angoff method to examine the progress test, which means that the calibration of the judges may not have been accurate. Thirdly, in our adaptation of the Angoff method, we did not perform an individual analysis on each judge’s estimations. Future development of this research should involve repetition of the proposed Angoff modification, to test its validity and reliability across different tests. Nonetheless, despite these limitations, our study demonstrated novel highlights regarding the better performance of items with high-level taxonomy, for obtaining better discrimination indices, and the high degree of precision of the panel of specialists regarding estimation of the difficulty of exam items.

Currently, item response theory is used to compose exams using previously tested items.^28^^,^^29^ Despite the advantages of this method, it has limited usefulness with regard to new written items. Our data suggest that classification of items using Bloom’s taxonomy (which can be performed prior to application of the exam) can select the items with better discrimination performance. Lastly, future research could provide correction formulas based on the judges’ expectations, in order to better predict the real difficulty of the items.

## CONCLUSION

In conclusion, the items with higher-level taxonomy provided better discrimination of the students’ performance; and the panel of experts demonstrated that they coherently deduced the difficulty of the exam items.
